# A Silent Threat: Tuberculous Pericarditis Presenting With Early Tamponade in a Young Migrant

**DOI:** 10.7759/cureus.97684

**Published:** 2025-11-24

**Authors:** Winluck Shayo, Rawan Manna, Julie Soo Fei Gan, Navid Munir, Surojit Bose

**Affiliations:** 1 Acute Medicine, University Hospitals of Derby and Burton NHS Foundation Trust, Derby, GBR; 2 Cardiology, University Hospitals of Derby and Burton NHS Foundation Trust, Derby, GBR

**Keywords:** cardiac tamponade, early diagnosis, echocardiography findings, extrapulmonary tuberculosis, migrant health, pericardial effusion, tuberculous pericarditis

## Abstract

Tuberculous pericarditis (TBP) is a rare but potentially life-threatening manifestation of extrapulmonary tuberculosis, particularly in regions with low tuberculosis (TB) prevalence. It accounts for a small fraction of pericardial effusion cases in high-income countries, yet remains a significant diagnostic challenge due to its nonspecific presentation and overlap with other infectious, inflammatory, or malignant causes. Here, we present a case of a tuberculous pericarditis in a 32-year-old male, originally from Congo, who presented with constitutional symptoms, pleuritic chest pain, and tachycardia shortly after migrating to the United Kingdom from South Africa. Initial investigations revealed a large pericardial effusion with early signs of tamponade, prompting urgent pericardiocentesis and further imaging. This case reinforces the need for timely recognition, multidisciplinary management, and public health awareness to mitigate the morbidity associated with tuberculous pericarditis.

## Introduction

Tuberculosis (TB), caused by Mycobacterium tuberculosis, remains one of the leading infectious causes of morbidity and mortality worldwide. While pulmonary TB is the most common manifestation, extrapulmonary involvement (including pericardial disease) can occur in up to 20% of cases, particularly in immunocompromised individuals or those from endemic regions [1]. Tuberculous pericarditis (TBP) is a rare but serious form of extrapulmonary TB, accounting for approximately 1-2% of all TB cases globally and up to 4% of pericarditis presentations in developed countries [2]. In contrast, in high-burden regions such as sub-Saharan Africa, TBP may account for 50-90% of effusive pericarditis cases [3].

The pathophysiology of TBP involves hematogenous or lymphatic spread of M. tuberculosis to the pericardium, resulting in granulomatous inflammation, exudative effusion, and potential progression to constrictive pericarditis. Clinical manifestations are often nonspecific, such as fever, chest pain, dyspnoea, and constitutional symptoms, making early diagnosis challenging [4]. Cardiac tamponade and constriction are life-threatening complications that require prompt recognition and intervention.

In the UK, TB incidence remains relatively low at 9.4 per 100,000, with 80% of cases occurring in individuals born outside the country [5]. Migration from endemic regions continues to shape the epidemiology of TB in high-income countries, necessitating vigilance for atypical presentations. This case report describes a young migrant presenting with large pericardial effusion and constitutional symptoms, ultimately diagnosed with tuberculous pericarditis. It highlights the importance of early imaging, pericardiocentesis, and histological confirmation. As well as the comparison between the diagnostic and therapeutic approaches and with the current literature.

## Case presentation

A 32-year-old male originally from Congo presented to the urgent treatment centre with a six-day history of worsening non-productive cough, pleuritic chest pain, fever, night sweats, and unintentional weight loss. He had recently returned from South Africa and travelled through multiple countries before arriving in the UK. He reported a prior BCG vaccination and a negative sputum TB test six months earlier. On examination, he was pyrexic (38.3°C), tachycardic (128 bpm), and normotensive. Respiratory rate was 17, and oxygen saturation was 100% on room air. Cardiovascular and respiratory examinations were unremarkable, with no signs of heart failure or chronic lung disease. The National Early Warning Score (NEWS) was four.

Initial labs (Table 1) demonstrated anemia (Hb 94 g/L), hypoalbuminemia (25-30 g/L), markedly raised C-reactive Protein (CRP) (91-110 mg/L), and elevated angiotensin-converting enzyme (ACE) (92 U/L), suggesting systemic inflammation and possible granulomatous disease. The markedly raised D-dimer (6876 ng/mL) was interpreted as a marker of inflammation rather than thromboembolism, given negative imaging for pulmonary embolism [6]. Pericardial fluid analysis revealed high lactate dehydrogenase (LDH) (399 U/L) and protein (58.7 g/L), consistent with an exudative effusion per Light’s criteria, supporting an infectious etiology. Echocardiography demonstrated right atrial collapse and respiratory variation in mitral inflow, indicating early tamponade physiology. Endobronchial ultrasound (EBUS) biopsy confirmed necrotizing granulomatous inflammation, strongly suggestive of Mycobacterium tuberculosis infection (Table 2). These findings collectively supported tuberculous pericarditis as the primary diagnosis.

**Table 1:** Comprehensive laboratory investigations including haematology, biochemistry, inflammatory markers, coagulation profile, immunoglobulin levels, and complement levels Hb: Hemoglobin; WBC: White Blood Cell count; Plt: Platelets; MCV: Mean Corpuscular Volume; ALP: Alkaline Phosphatase; ALT: Alanine Aminotransferase; GGT: Gamma-Glutamyl Transferase; LDH: Lactate Dehydrogenase; CRP: C-reactive Protein; ACE: Angiotensin-Converting Enzyme; PT: Prothrombin Time; INR: International Normalized Ratio; APTT: Activated Partial Thromboplastin Time; Ig: Immunoglobulin; C3/C4: Complement components.

Parameter	Day 0	Day 2	Reference Range
Haematology			
Hb (g/L)	94	103	130–170
WBC (×10⁹/L)	7.2	7.0	4–10
Platelets (×10⁹/L)	356	395	150–410
MCV (fL)	85.3	85.8	83–101
Biochemistry			
Bilirubin (µmol/L)	13	14	0–21
ALP (U/L)	132	116	40–129
ALT (U/L)	47	42	0–40
Albumin (g/L)	30	25	35–50
GGT (U/L)	153	—	8–60
LDH (blood) (U/L)	237	—	0–250
Inflammatory Markers			
CRP (mg/L)	91	110	0–5
ACE (U/L)	92	—	20–70
Coagulation Profile			
PT (s)	13	—	9.2–12
INR	1.16	—	0.9–1.1
APTT (s)	33.3	—	20–30
Fibrinogen (g/L)	5.04	—	1.5–4.7
Other			
D-Dimer (ng/mL)	6876	—	0–500
Troponin T (ng/L)	<10	—	0–13
Immunology			
IgG (g/L)	14.41	—	6–16
IgM (g/L)	0.18	—	0.5–2
IgA (g/L)	2.43	—	0.8–4
Complement C3 (g/L)	1.7	—	0.9–1.8
Complement C4 (g/L)	0.67	—	0.1–0.4

**Table 2:** Tuberculosis diagnostic panel and pericardial fluid analysis findings AFB: Acid-Fast Bacilli; PCR: Polymerase Chain Reaction; EBUS FNA: Endobronchial Ultrasound-Guided Fine Needle Aspiration; LDH: Lactate Dehydrogenase; TP: Total Protein

Test	Result
Acid-fast bacilli (AFB)	Not seen
Mycobacterial culture	Mycobacterium tuberculosis isolated
PCR	Negative on admission; positive at 2 weeks
EBUS FNA	Necrotising granulomatous inflammation
LDH (pericardial fluid) (U/L)	399
Total Protein (g/L)	58.7

**Figure 1:**
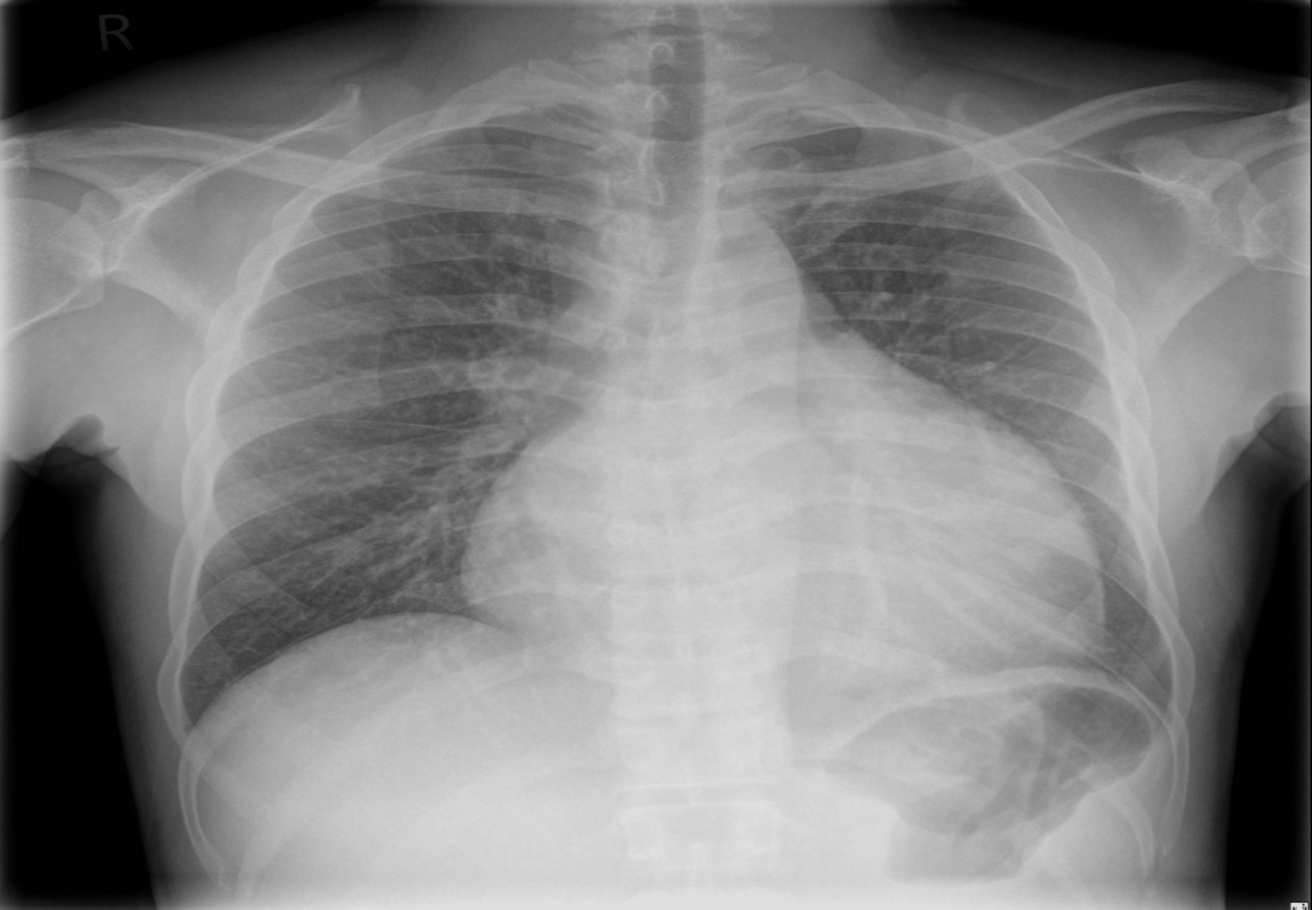
Chest X-Ray Frontal chest radiograph demonstrates an enlarged cardiac silhouette, suggestive of pericardial effusion. A small amount of fluid is visible in the horizontal fissure. No pulmonary infiltrates or cavitary lesions are identified.

**Figure 2:**
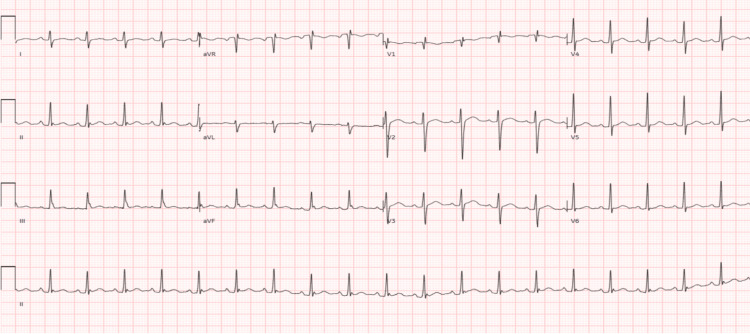
Electrocardiogram Admission ECG shows sinus tachycardia at 128 bpm without ST-segment elevation, electrical alternans, or classic pericarditis changes. Follow-up tracings demonstrated T-wave inversions in lateral leads (I, II, aVL, V5, V6), consistent with pericardial irritation.

**Figure 3:**
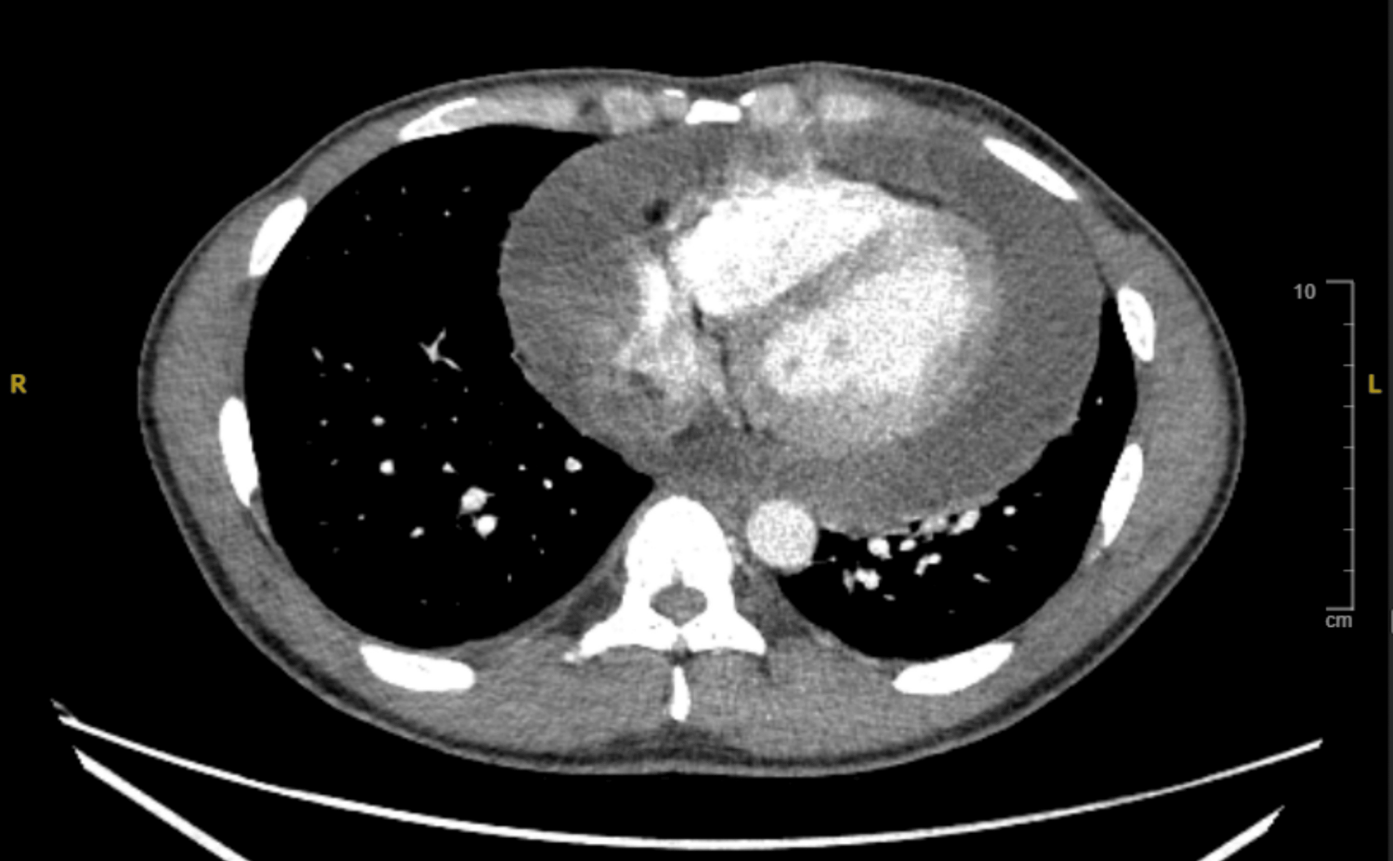
CT Thorax Contrast-enhanced CT thorax illustrating pericardial effusion and mediastinal lymphadenopathy.

Chest X-ray demonstrated cardiomegaly with a trace of pleural fluid in the horizontal fissure (Figure 1), raising suspicion for pericardial effusion. ECG (Figure 2) showed sinus tachycardia and later T-wave inversions in lateral leads (I, II, aVL, V5, V6), suggestive of pericardial irritation. Transthoracic echocardiography confirmed a large pericardial effusion with early tamponade physiology, evidenced by right atrial collapse and respiratory variation in mitral inflow (Figure 3), findings consistent with tuberculous pericarditis described in the literature [7].

Contrast-enhanced CT thorax confirmed a moderate to large pericardial effusion and mediastinal lymphadenopathy, with the largest right paratracheal node measuring 2.5 cm (Figure 3). Endobronchial ultrasound-guided biopsy of these nodes revealed extensive necrotizing granulomatous inflammation, strongly suggestive of Mycobacterium tuberculosis infection (Table 1). EBUS is recognized as a minimally invasive and highly sensitive technique for diagnosing mediastinal tuberculosis, particularly when pericardial fluid analysis is inconclusive [8].

The patient was admitted to the coronary care unit and underwent emergency pericardiocentesis, draining approximately 900 mL of blood-stained fluid. This intervention was both diagnostic and therapeutic, improving hemodynamics and reducing the risk of tamponade, as supported by previous studies [9].

He was initiated on colchicine and ibuprofen for pericardial inflammation, in line with European Society of Cardiology guidelines [10]. Following microbiological confirmation, standard anti-tuberculous therapy (rifampicin, isoniazid, pyrazinamide, and ethambutol) was commenced. Repeat imaging prior to discharge showed complete resolution of the pericardial effusion and stable mediastinal lymphadenopathy.

The patient was discharged with scheduled follow-up in the TB clinic and cardiology services every three months for the first year. Colchicine was continued for three months, ibuprofen for two weeks, and nutritional support was provided by the dietetics team.

## Discussion

Tuberculous pericarditis remains a diagnostic challenge due to its nonspecific symptoms and overlap with other causes of pericardial effusion, including viral, autoimmune, and malignant aetiologies [11]. In this case, the patient’s migratory history and constitutional symptoms prompted early consideration of TB, despite the absence of pulmonary involvement, a pattern increasingly reported in high-income countries [12].

Imaging played a pivotal role in diagnosis. Echocardiography revealed early tamponade physiology, while CT thorax identified lymphadenopathy suggestive of granulomatous disease. These findings align with recent studies advocating multimodal imaging for TBP evaluation [13]. EBUS-guided biopsy provided histological confirmation, a technique shown to outperform pericardial fluid analysis in sensitivity and specificity [8].

Pericardiocentesis was both diagnostic and therapeutic. While some studies advocate pericardial window for recurrent effusions, early percutaneous drainage remains the preferred initial approach in hemodynamically stable patients [14]. Adjunctive anti-inflammatory therapy with colchicine and NSAIDs is supported by guidelines, though corticosteroids remain controversial due to mixed evidence on their impact in HIV-negative patients [15].

Compared to other cases, this patient benefited from early intervention before the onset of overt tamponade or constriction. A case reported by Yousif et al. described a patient progressing to constrictive pericarditis despite anti-TB therapy, requiring surgical pericardiectomy [16]. This underscores the importance of timely drainage and close follow-up.

The absence of HIV co-infection in our patient likely contributed to his good prognosis. HIV-positive individuals have higher rates of TBP and worse prognosis, with studies showing increased mortality and risk of constriction [17]. In the context of global migration and rising TB incidence among foreign-born individuals in low-prevalence countries, clinicians must remain vigilant for extrapulmonary manifestations of tuberculosis.

## Conclusions

This case emphasizes the importance of maintaining a broad differential diagnosis when evaluating pericardial effusion, especially in patients from TB-endemic regions. It also highlights the utility of multimodal imaging and histological confirmation via EBUS in establishing a definitive diagnosis.

Early echocardiography identified tamponade physiology, prompting urgent pericardiocentesis and preventing hemodynamic collapse. EBUS-guided biopsy provided a definitive diagnosis when fluid analysis was inconclusive, enabling the timely initiation of anti-TB therapy and avoiding progression to constrictive pericarditis.

Clinicians should maintain a high index of suspicion for TB in atypical presentations, even in low-incidence settings. Prompt initiation of anti-tuberculous therapy and multidisciplinary care can prevent complications and improve outcomes.
